# Supplementary material for: Chromothripsis during telomere crisis is independent of NHEJ, and consistent with a replicative origin
Source: Genome Res. 2019 May;29(5):737–49. doi: 10.1101/gr.240705.118 (PMC6499312; doi:10.1101/gr.240705.118)
Supplement: Supplemental Material [file supp_gr.240705.118_Supplemental_file_1.zip › contigs/annotated_contigs/DB57/contig.2.DB57_length_677_mean_cov_7.80059084195.docx]

**DB57_length_677_mean_cov_7.80059084195**

```
AAGCCATCAGGATGATAAGGACCTAACATACAAATCTACTCTGAGGCTGTGGTTCTTAGCCCTAGGTGAGCATCAGAACTACTCATAGA
 >chr17:46287512-46287832 - E=9e-175
ACTTTGTAAAAATAAACATGCCTCAGCCCCACTCCAGATAATCCTGAATCAGTATATCTGGGGTGCAGTTCAGGTATCTACACTTTTTT

AAAAAGTAGACAGGTGATTCCGATGTGCAGCCAGAGTTACGAACCACGGCTCCAAGGTGTCGATGACTGATAATGGCTCCGTATAATAG

TGCCAGCCACACACACGTGACCTCGTGGATAAGCGTCGCTACCTGACATCTGT|ATT|CCAAGGCTGGAATACAGTAGCGCCATCATAG
                                                         >chr17:46265671-46266025 - E=3
CTCACTGCAGCCTCCAATCCTGGGCTCAAGCAATGCTCCCACTTCAGCCTGTCAAAGAGCTGAGACAACAGGTGCACACAACTGTGCCT
e-201
GGCTATTTTATTATTTTTTTATAGAGACAAGGTCTCACTTTGTTGCCCAGGCTGGTCTCAAACTCCTGGCTTTAAGCATACTTCCCATC

TCAGCCTCCCAAAGTGCTGGGATTACAGATGTGAGCCACCACTTCTGCCTTCAAACCCAACTCTTGACCTTTAACTCATTTAATTAGAG

TAGGTACCACTAGAGTTGGCATCTCAGGTTTAACACTCAACAGGCAAATTGGCTCA
```
